# Multiple Anterior and Posterior Circulation Intracranial Fenestrations presenting With Aneurysmal Subarachnoid Hemorrhage

**DOI:** 10.7759/cureus.12667

**Published:** 2021-01-12

**Authors:** Robert A Scranton, Amanda V Jenson, Rishi Suresh, Gavin Britz

**Affiliations:** 1 Neurosurgery, Goodman Campbell Brain and Spine, Indianapolis, USA; 2 Neurosurgery, Houston Methodist Neurological Institute, Houston, USA; 3 Neurological Surgery, Texas A&M College of Medicine, Bryan, USA; 4 Neurological Surgery, Houston Methodist Hospital, Houston, USA

**Keywords:** fenestration, cerebral aneurysm, subarachnoid hemorrhage

## Abstract

*Background and Importance*: Intracranial artery fenestrations are very rare, however, when found, there is a high association with cerebral aneurysms.

*Clinical Presentation*: This report describes a patient with multiple anterior and posterior circulation intracranial artery fenestrations and an anterior communicating artery aneurysm presenting with a thunderclap headache found to have a subarachnoid hemorrhage (SAH). The patient was treated with open surgery via clipping after a diagnostic angiography and did very well.

*Conclusion*: There is an association between cerebral fenestrations and aneurysms, but it has not been studied in a prospective manner. This case is unusual in that the patient had both anterior and posterior circulation fenestrations, which is uncommon. Clinicians should have a high index of suspicion in patients being evaluated for SAH who have a cerebral artery fenestration with no aneurysm found.

## Introduction

Arterial fenestrations are known variants, where vessels divide into separate channels with distinct endothelial and muscle layers, which then reform into a single channel. Single and multiple fenestrations have been described [1-2]. Intracranial artery fenestrations are rare, but closely associated with aneurysms. Cooke et al. reviewed 11,000 cases and reported an intracranial artery fenestration prevalence of 2.1%, where 60.5% of patients harbored at minimum one aneurysm and 52% of patients with both an aneurysm and fenestration presented with subarachnoid hemorrhage (SAH) [3]. The precise number is unknown, given the retrospective nature and selection bias of most series. We present a case of aneurysmal SAH in a patient with multiple anterior and posterior circulation intracranial fenestrations.

## Case presentation

The patient is a 45-year-old male who presented to an outlying emergency department complaining of the worst headache of his life, neck pain, and nausea. Further patient history included human immunodeficiency virus (HIV), hypertension, and tobacco abuse with a 25 pack-year history. He was found to be hypertensive on arrival, for which he was treated and discharged with blood pressure and pain medication to follow-up with his primary care provider. No cranial imaging or lumbar puncture was performed. The patient then called emergency medical services (EMS) to home the same day of discharge and was taken to another facility with similar complaints of intense headache associated with nausea. After receiving treatment for his blood pressure of 210/120 mmHg, a non-contrast CT of his head was performed and an SAH was diagnosed.

**Figure 1:**
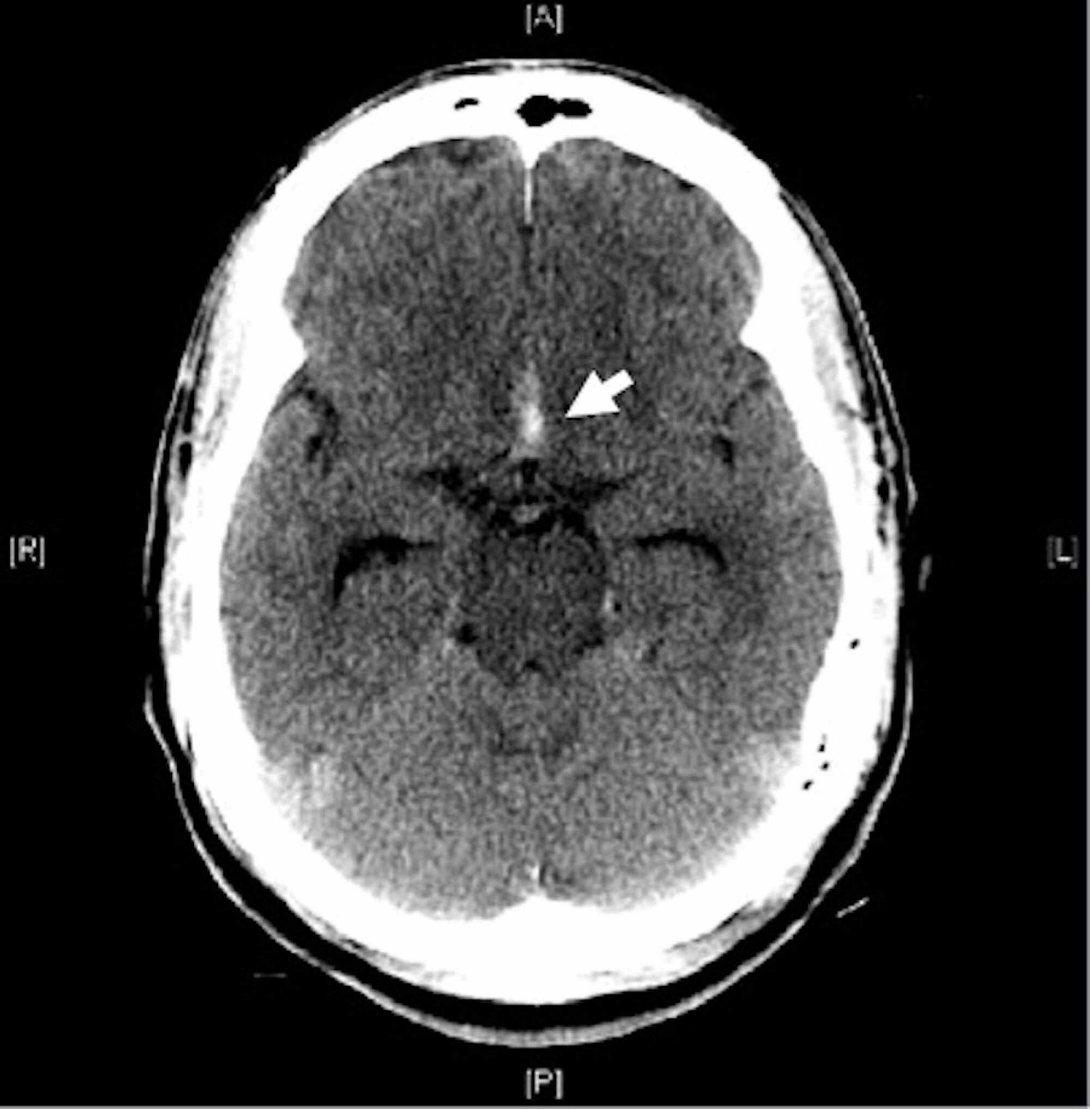
Axial non contrasted CT of the brain showing subarachnoid hemorrhage in interhemispheric fissure (arrow).

The patient was then transferred to our facility. The review of his CT scan (Figure 1) revealed blood in the interhemispheric fissure suspicious for aneurysmal SAH.

**Figure 2:**
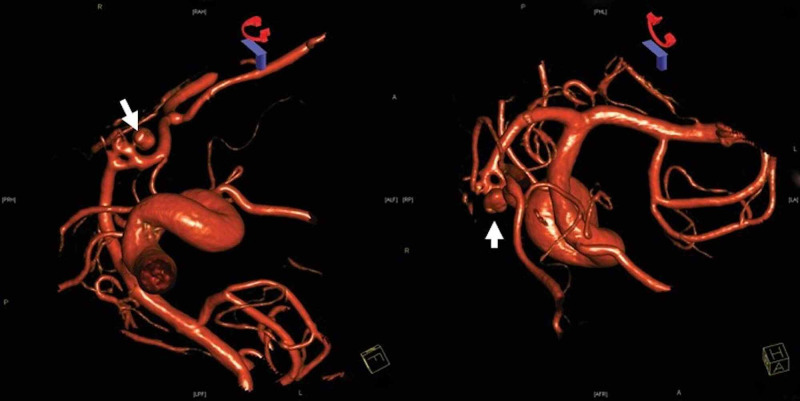
Digital subtraction cerebral angiography with 3D reconstruction showing fenestration of the anterior communicating complex associated with a 5 mm aneurysm (arrows)

**Figure 3:**
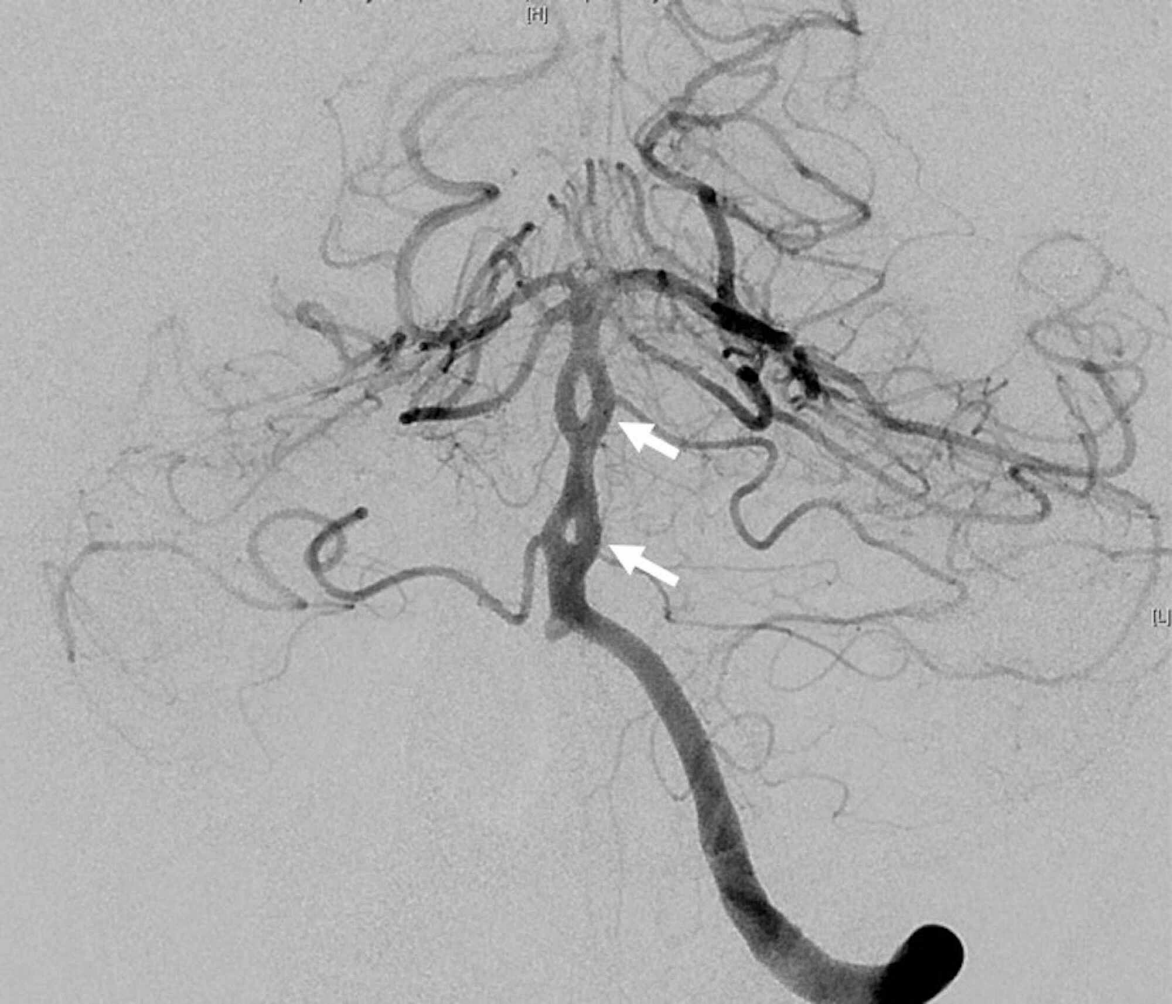
Digital subtraction cerebral angiography of posterior circulation showing a double fenestration of the basilar artery (arrows)

Digital subtraction cerebral angiography with 3D reconstruction was performed and a fenestration of the anterior communicating complex was found, associated with a 5 mm aneurysm (Figure 2). There was also a double fenestration of the basilar artery (Figure 3). The location of the aneurysm, arising from a fenestrated anterior communicating complex, made the endovascular approach difficult, therefore, open treatment was recommended. The patient was taken to the operating room and a left-sided frontal-temporal approach was used. The aneurysm appeared to have a narrow neck on the 3D reconstruction, however, it was in fact wide-necked. A fenestrated clip was used to preserve the ipsilateral A1 while excluding the aneurysm from circulation. Intraoperative angiography confirmed the exclusion of the aneurysm with the preservation of branches. The patient was treated for symptomatic vasospasm post-bleed for days 4 through 14 and discharged home on postoperative day 16.

## Discussion

The posterior fossa is the most common location for intracranial artery fenestrations, usually in the basilar artery followed by the vertebral. A fenestration in the anterior circulation is usually associated with the anterior communicating complex. Multiple fenestrations are exceptionally rare, only found in 2.2% of patients with fenestrations [3].

Several mechanisms have been described to explain the development of arterial fenestrations in relation to cerebral vascular embryogenesis, classically described by Padget [4]. The anterior circulation forms primarily from the cerebral branch of the internal carotid, a vascular network forms between the paired cerebral branches that then regresses [5]. Errors in this network formation and regression are the basis of the plexiform theory for fenestration formation that explains the origin of anterior communicating complex fenestrations. In the posterior circulation, the basilar artery forms from the paired longitudinal dorsal arteries and fusion of the ascending ramus of the anterior radicular arteries. Incomplete fusion is the likely mechanism resulting in basilar fenestrations [6].

The patient presented here harbored a double fenestration of the basilar artery as well as a single fenestration of the anterior communicating complex that was associated with a ruptured aneurysm. It is unclear whether there is an association between aneurysms and arterial fenestrations.

The possible association has been described in two contexts: aneurysms remote to the fenestration, or aneurysms associated with the fenestration. One theory reports that fenestrations may be a general marker of diseased vasculature and a high prevalence of remote aneurysms would be expected. Published series to date do not support this conclusion and suggest that this would be contrary to theories regarding the embryological origin of fenestrations. However, one could consider that if fenestrations are of embryological origin, the presence and development of aneurysms could be favored by some altered vessel wall, and therefore, develop later during life. It is known that altered flow dynamics in the presence of fenestrations may promote aneurysm development, though the exact relationship is not well known and the embryological origin is controversial.

Aneurysms may be seen at fenestration branch points. However, in our patient, the aneurysm arose from the long segment of the anterior communicating complex fenestration rather than a branch point. The microanatomy of fenestration branch points has been studied and defects of the intima and media layers can be found at both proximal and distal branch points [7]. Additionally, fenestrations may share an adventitial layer or have separate layers. Media defects at branch points may predispose to aneurysm formation, a common feature observed at natural branch points as well [8]. Whether a fenestration branch point is more susceptible to aneurysm formation in comparison to a natural branch point is unclear. Series to date are retrospective in nature and subject to a great deal of selection bias. The largest series available, from the group in San Francisco, examined almost 11,000 digital subtraction angiograms (DSAs) reports rather than a review of the images [3]. The authors noted the study may underreport the incidence of fenestrations as they are considered a known variant rather than pathologic and may not merit comment in the report. Similar to other cohorts based on some form of cranial imaging (DSA, computed tomography angiogram [CTA], and magnetic resonance angiogram [MRA]), the population is drawn from patients who had a clinical situation warranting imaging creating a significant selection bias. The impact of these limitations may be negated somewhat with large sample sizes, as such we believe a prevalence of 2.1% is likely the best estimate possible. However, the selection bias in the retrospective series can not be discounted and a relation between arterial fenestration and aneurysm formation remains purely speculative.

## Conclusions

Intracranial aneurysms may be seen in up to 60% of patients with intracranial fenestrations. Insufficient evidence exists to determine if this represents a causative relationship or is coincidental. Patients with fenestrations should receive extra scrutiny when being evaluated for aneurysms. Future studies are needed to determine if this population of patients are at a higher risk for SAH or de novo aneurysm formation.
